# Different living environments drive deterministic microbial community assemblages in the gut of Alpine musk deer (*Moschus chrysogaster*)

**DOI:** 10.3389/fmicb.2022.1108405

**Published:** 2023-01-13

**Authors:** Zhirong Zhang, Mengqi Ding, Yujiao Sun, Romaan Hayat Khattak, Junda Chen, Liwei Teng, Zhensheng Liu

**Affiliations:** ^1^College of Wildlife and Protected Areas, Northeast Forestry University, Harbin, China; ^2^State Key Laboratory of Urban Water Resource and Environment, School of Environment, Harbin Institute of Technology, Harbin, China; ^3^College of Food and Biological Engineering, Henan University of Animal Husbandry and Economy, Zhengzhou, China; ^4^Key Laboratory of Conservation Biology, National Forestry and Grassland Administration, Harbin, China

**Keywords:** ruminants, microbial diversity, assembly mechanism, environmental variation, function

## Abstract

Substantial variation in the environment directly causes remodeling of the colonized gut microbiota, controlling community diversity, and functions in the host to tune-up their adaptive states. However, the mechanisms of microbial community assembly in response to environmental changes remain unclear, especially in endangered ruminants. In this study, we analyzed the microbial communities of 37 fecal samples collected from captive and wild Alpine musk deer (*Moschus chrysogaster*) to characterize the complexity and assembly processes using 16S rRNA gene sequencing. We found significantly different diversities and compositions of gut microbiota among both groups associated with different living environments. Heterogeneous selection was the predominant factor regulating the gut microbiota community under similar climatic conditions, indicating that microbial community assembly was largely driven by deterministic mechanisms. The species co-occurrence network showed complex and tight connections with a higher positive correlation in the wild environment. Moreover, the captive group exhibited significant differences in chemoheterotrophy and fermentation compared with the wild group, but the opposite was observed in animal parasites or symbionts, which might be closely related to diet, energy supply, and healthcare of animals. This study provides a framework basis and new insights into understanding gut microbiota in different environments.

## 1. Introduction

Microbial communities that colonize the gastrointestinal tract at birth are critical in maintaining the health of the host (Ben Shabat et al., 2016; Zeevi et al., 2019; Deng et al., 2022) and can be shaped with diet (David et al., 2014; Schnorr et al., 2014; Friedman et al., 2017), phylogenic development (Turnbaugh et al., 2009; Jiang et al., 2021), and environment modifications (Wang et al., 2019; Zhang et al., 2022). Changing and shaping of gut microbiota are particularly apparent in captive and translocated populations (Li et al., 2017; Sun et al., 2020), indicating the manifestation of co-evolution and adaptation to the external environment between a host and its microbial communities. This process provides an optimal opportunity to understand microbial diversity, assembly patterns, and complex connection(s) to function.

Understanding the microbial community assembly mechanism is a key topic in microbial ecology and is an objective method that can facilitate obtaining health data and preventing potential disease in animals (Nemergut et al., 2013). It is commonly debated that deterministic processes, including biotic (interspecies interactions such as competition, predation, and mutualism) and abiotic factors (environmental filtering such as pH, temperature, and salinity), and stochastic processes (e.g., dispersal, immigration, speciation, and drift) are affected by the assembly of microbial communities (Chesson, 2000; Fargione et al., 2003; Chave, 2004; Wang et al., 2013; Zhou and Ning, 2017). Previous studies on microbial community assembly have predominantly focused on environment-related factors (Jiao et al., 2019; Chen et al., 2022) and hydrobionts (Kokou et al., 2019; Mo et al., 2021; Liu et al., 2022), and there has been a lack of studies in mammals, especially rare and endangered species.

Musk deer (*Moschus* spp.), a small and timid ruminant, is widely known for its musk secreted by males (Meng et al., 2011). For this reason, this population has historically suffered severe disasters (poaching and illegal trade), resulting in a dramatic decline in the population size, which has remained at a low level. Alpine musk deer (*Moschus chrysogaster*), the largest musk deer in terms of body size, lives in a narrow area bordering Bhutan, India, Nepal, and China (Harris, 2016). In addition, local isolated populations of Alpine musk deer exist in the Helan Mountains, separated by the Yellow River, the desert, and the city, on the edge of the Qinghai–Tibet Plateau in China; however their population sizes are not promising. Moreover, the population is far less competitive than other sympatric ruminants [e.g., blue sheep (*Pseudois nayaur alashanicus*) and red deer (*Cervus elaphus alashanicus*)] and is very sensitive to disturbances. Alpine musk deer is listed as an endangered species on the Red List by the International Union for Conservation of Nature (IUCN) and is a national key protected wildlife of class I in China (Jiang et al., 2020). Small populations of Alpine musk deer are present in captivity in China. Musk deer are susceptible to gastrointestinal diseases in captivity owing to frequent changes in diet, unclean water, transmission of germs carried by keepers, changeable weather, and so on. However, limited food with disparate sources is the major factor between captive and wild musk deer that directly affects and shapes the gut microbiota associated with relevant functions to adapt to change (Li et al., 2017; Sun et al., 2020). Clarification of the assembly processes of the microbial community of musk deer under different feeding conditions is needed to facilitate population conservation and restoration.

In our previous study investigating the gut microbiota of captive (C) and wild Alpine musk deer (W), different microbiota were enriched (C: Firmicutes; W: Proteobacteria, and Euryarchaeota) to link with different functions and metabolic pathways (C: methanogenesis; W: digesting cellulose, and generating short-chain fatty acids) (Sun et al., 2020). However, gut microbiota assembly processes related to the aforementioned differences are still unclear. In this study, metadata of gut microbiota were collected, and null modeling and network analysis were conducted to estimate the relative importance of ecological processes and reveal complex connections and functional predictions between captive and wild Alpine musk deer. The objectives of the study were to (a) compare the diversity and composition of gut microbiota of C and W groups, (b) elucidate the mechanisms of microbial community assembly processes in this context, and (c) analyze the interaction(s) among indicators and predict potential functions. This study provides a framework basis and new insights into understanding gut microbiota in different environments.

## 2. Material and methods

### 2.1. Sample collection and sequencing

In total, 14 fecal samples were collected from the Xinglongshan Alpine musk deer farm in Lanzhou city, Gansu Province (104°6′E, 35°48′N). Prior to the collection of the samples, the animals were healthy, no diseases were observed, and they had been domesticated and bred for over 30 years. A total of 23 fecal samples were collected from wild Alpine musk deer at the Helanshan National Nature Reserve in the Inner Mongolia Autonomous Region, China (105°48′-57′E, 38°39′-51′N). All samples were collected around February 2015 as they were less susceptible to deterioration at the low temperatures of this time of year. All feces were pellet-like without diarrhea and were immediately stored at −80°C. Total DNA from fecal samples was extracted using the QIAamp Fast DNA Stool Mini Kit (QIAGEN, Hilden, Germany) following the manufacturer's protocol. DNA was diluted to 1 ng/μL. The V3–V4 hypervariable region of the 16S rRNA gene was amplified with specific primers (515F: 5′-GTGCCAGCMGCCGCGGTAA-3′; 806R: 5′-GACTACHVGGGTWTCTAAT-3′). The PCR system contained 1.5 μL of DNA template, 10 μL of Phusion High-Fidelity buffer (5×), 0.5 μL of Phusion DNA polymerase, 1 μL (10 mM) of dNTPs, 0.5 μM of forward and reverse primers, and ddH_2_O of 50 μL. The PCR conditions comprised an initial denaturation at 98°C for 30 s, denaturation at 98°C for 5–10 s, annealing at 55°C for 30 s, 35 cycles of elongation at 72°C for 3 min, and a final elongation at 72°C for 20 min. Next, 16S rRNA libraries were constructed using a TruSeq DNA PCR-Free Sample Preparation Kit (Illumina, United States) and sequenced on an Illumina HiSeq platform (HiSeq 2500, PE250). Sequence data were mainly analyzed on the QIIME platform (v1.9.1), and chimeras were filtered and removed by using FLASH (v1.2.11). Sequences with an identity threshold of 0.97 were defined as the same operational taxonomic units (OTUs) using UPARSE (v7.0.1090). Taxonomy of the OTUs was performed by annotation using Ribosomal Database Project (RDP) (v11.5) against the Silva 138 16S rRNA database at a confidence threshold of 70%.

### 2.2. Environment-related microbial community structure analysis

Alpha diversity (including the Sobs, Ace, Simpson, Chao1, and Smith–Wilson indices) was calculated by mothur (v1.30.2). Non-metric multidimensional scaling (NMDS) based on Bray–Curtis dissimilarities was performed for each microbiome by calculating compositional similarity and mapping using the online Majorbio Cloud platform.^1^

Partial least squares–discriminant analysis (PLS-DA), a supervised discriminant analysis method, was conducted to cluster microbial data based on their sources (Metwaly et al., 2020). To assess the relative importance of deterministic and stochastic processes on microbial community assembly, the null model analysis was first applied, and 999 randomizations were used to generate model expectations. The β-nearest taxon index (βNTI) and the Bray–Curtis-based Raup–Crick (RCBray) index were used to measure the variation in both phylogenetic and taxonomic diversities (Stegen et al., 2013). Then, Levins' niche width index was applied to estimate the patterns of determinism and stochasticity and their influence on microbial communities (Levins, 1968; Finn et al., 2020). Meanwhile, according to the community structure distribution of samples among groups, source tracking, which predicts the composition proportion, was performed based on Bayesian algorithms (Knights et al., 2011).

Random forest, a tree-based machine-learning model, was applied to examine the clustering of each sample and calculate variable importance using mean decrease accuracy (Boulesteix et al., 2012; Pawlik and Harrison, 2022; Zhao et al., 2022). The area under the curve (AUC) was used to evaluate the accuracy of the model, and data were normalized using Z-score. To identify the characteristics and categories of the gut microbiota between all groups, the Indval function in the “labdsv” R package was used to seek indicators showing significant differences (Ren et al., 2022). All indicators with a significant difference were classified into three levels based on the relative abundance, with thresholds of 0.75 (high), 0.50 (medium), and 0.25 (low), and indicators with a low relative abundance that cannot be ignored were defined as rare species. A network analysis based on Spearman's correlations (|*r*| > 0.5, *p* < 0.05) was performed to fully describe the covariation between captive and wild groups. The top 100 abundant OTUs, which are considered to play important roles and show complex positive and negative interactions in the microbial community, were identified based on the topological roles of their module-based co-occurrence networks (Deng et al., 2012).

To predict the differences in microbial phenotypic information between the two groups, the Wilcoxon rank-sum test was conducted on the BugBase cloud platform^2^ (Zhang et al., 2019). Functional annotation of prokaryotic taxa (FAPROTAX) was used to predict microbial functions. FAPROTAX predictions were based on the normalized contig-based 16S rRNA OTU table annotated in the database of prokaryotic environmental functions (Louca et al., 2016).

### 2.3. Statistical analysis

All reported values are expressed as mean ± standard deviation (SD). Differences in microbial diversity, niche width, phenotype, and predicted function between groups were analyzed by using the Wilcoxon rank-sum test and visualized using Origin (v2023). Annotations with a corresponding sign in the text were considered statistically significant.

## 3. Results

### 3.1. Gut microbiota of Alpine musk deer are environment-specific

**Figure 1:**
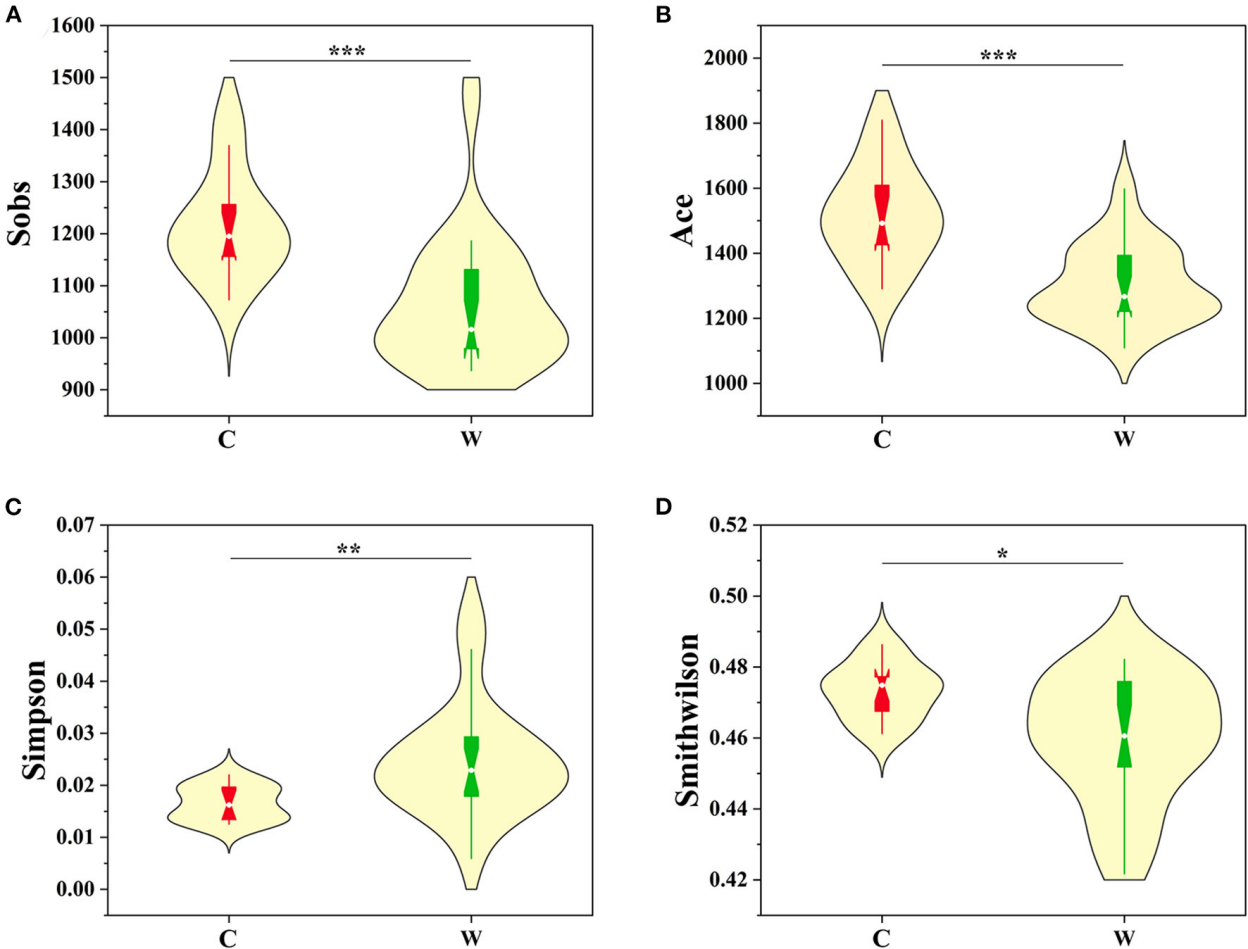
Comparison of alpha diversity indices [**(A)** Sobs, **(B)** Ace, **(C)** Simpson, and **(D)** Smithwilson] of gut microbiota of the captive (C) and wild (W) Alpine musk deer (significance *p < 0.05, **p < 0.01, and ***p < 0.001).

Environment specificity of Alpine musk deer microbes was assessed by comparing the similarities of microbial communities of hosts in captivity (*n* = 14) and those in the wild (*n* = 23). Alpha diversity (Sobs, Ace, Simpson, Chao1, and Smith–Wilson indices) and beta diversity were used to evaluate the structure of gut microbiota in captive (C) and wild (W) Alpine musk deer. The Sobs Index values in C and W Alpine musk deer were 1,217.20 ± 102.18 and 1,063.20 ± 127.24, respectively (Figure 1A); the Ace Index values in the C and W groups were 1,519.00 ±142.59 and 1,303.40 ±121.43, respectively (Figure 1B); the Simpson Index values in the C and W groups were 0.017 ± 0.003 and 0.025 ± 0.011, respectively (Figure 1C); the Smith–Wilson values in the C and W groups were 0.473 ± 0.008 and 0.459 ± 0.033, respectively (Figure 1D); and the Chao1 Index values in the C and W groups were 1,531.40 ±142.59 and 1,311.50 ± 123.33, respectively (Supplementary Figure 1). All of these indices were significantly higher in the captive Alpine musk deer group than in the wild group (*p* < 0.01), which indicated that there was a significant difference associated with the living environments of Alpine musk deer. Furthermore, NMDS on OTU level based on Bray–Curtis dissimilarities revealed a clear cluster of the gut microbial communities from the captive group and the wild group, respectively (Supplementary Figure 2A), and a cluster between females (C–F) and males (C–M) in the captive group (Supplementary Figure 2B).

### 3.2. Microbial community assembly processes are relatively deterministic

**Figure 2:**
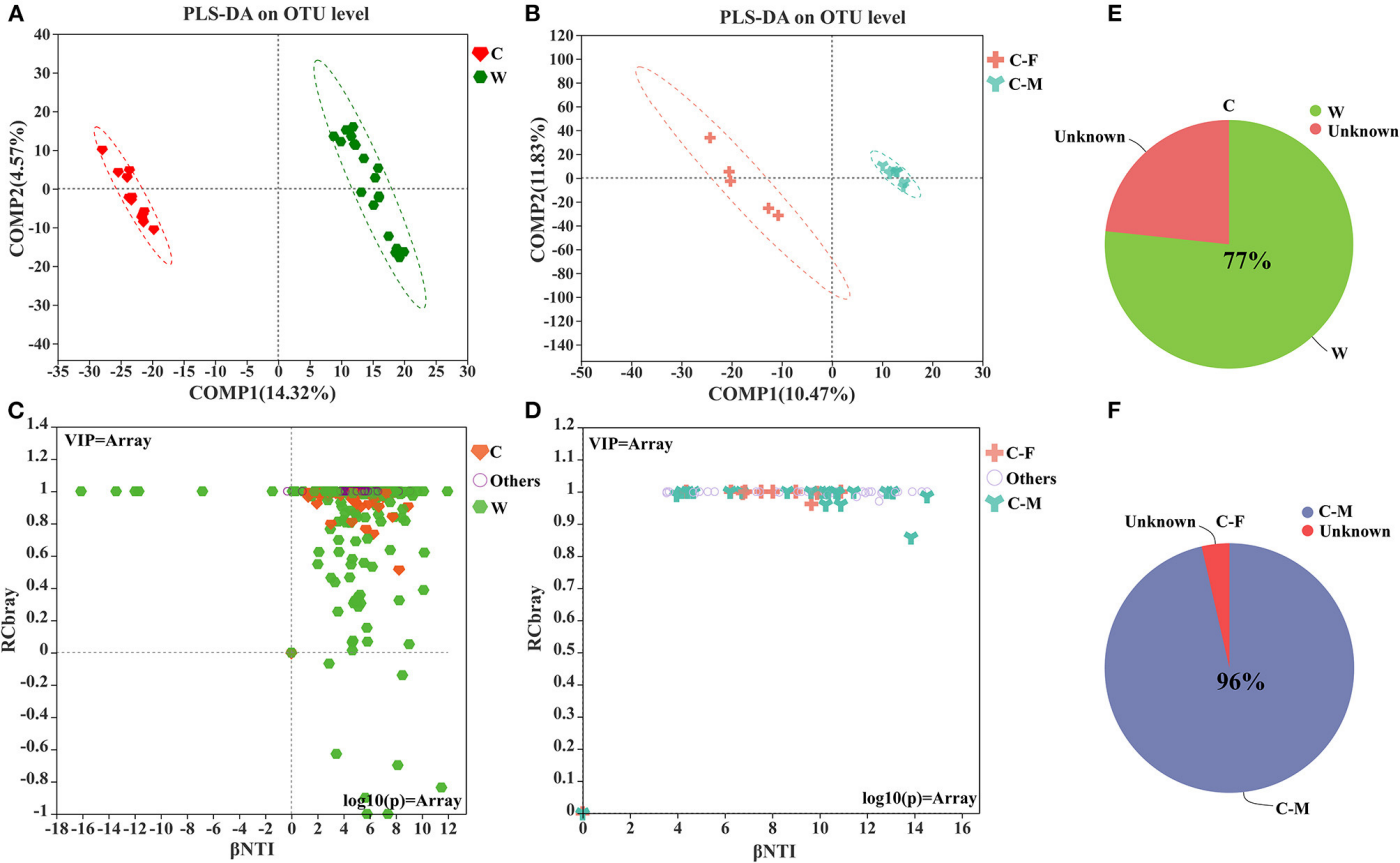
**(A, B)** Partial least squares-discriminant analysis (PLS-DA) of microbial communities for each group; **(C, D)** Microbial community assembly or community structure by βNTI/RCbray; **(E, F)** Pie charts of the mean SourceTracker proportion estimates for 100 draws from Gibbs sampling in captive vs. wild groups and captivity between female and male groups (W: wild, C: captive, C-F: captive female, and C-M: captive male).

**Figure 3:**
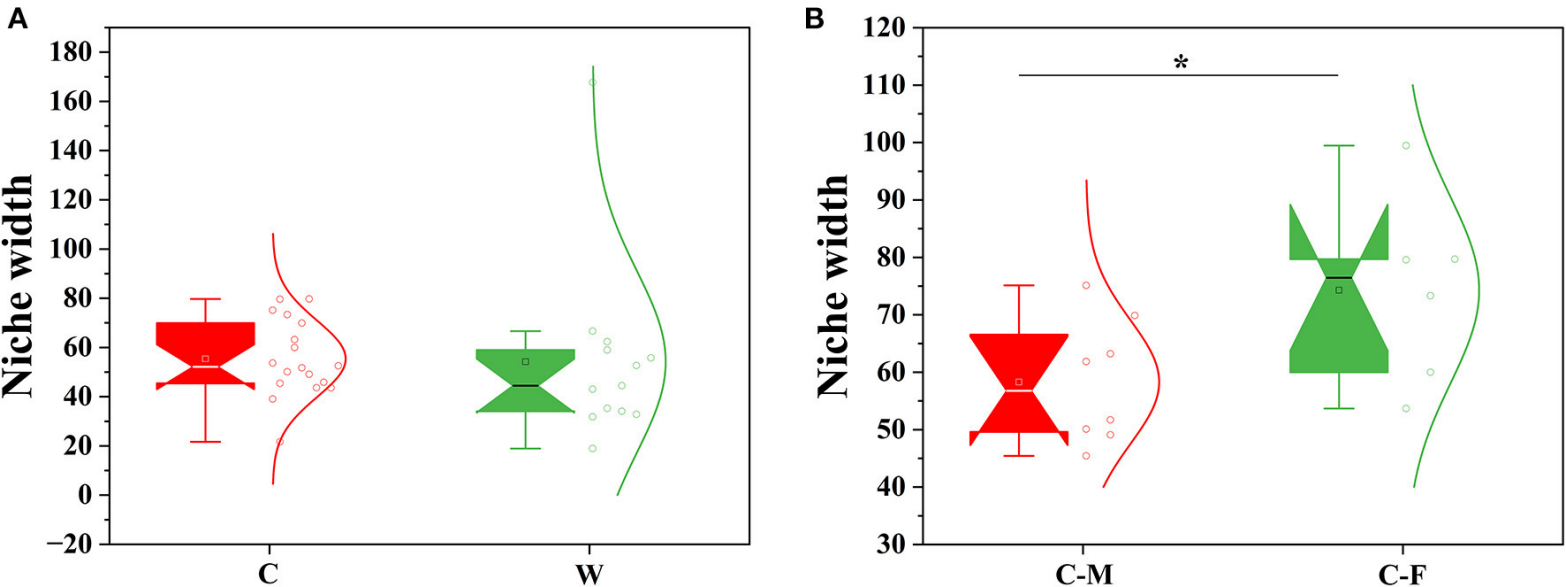
Comparison of mean habitat niche width for all groups. **(A)** Wild vs. captive groups. **(B)** Captive female vs. captive male groups (W: wild, C: captive, C-F: captive female, and C-M: captive male; Significance *p < 0.5).

The PLS-DA score plot on the OTU level further showed that samples clustered together according to different living environments and confirmed the gender-related clustering phenomenon in captive Alpine musk deer by applying this supervised cluster analysis method (Figures 2A, B). The null model analysis also showed a higher relative contribution of deterministic processes (|βNTI| ≥ 2) in Alpine musk deer gut microbiome assembly was largely affected by different living environments and sexes (Figures 2C, D). Furthermore, the niche width calculated based on the OTU level was estimated to display the contributions of species classification and dispersal limitation to microbial community construction. The niche width values in the C and W groups were 55.40 ± 15.17 and 54.20 ± 35.43, respectively, and no significant difference was observed between the two groups (*p* > 0.05, Figure 3A), while there was a significant difference in the niche width between C–F (74.29 ± 14.84) and C–M groups (58.30 ± 10.79; *p* < 0.05; Figure 3B). Moreover, 77% of the gut microbiome of the C group was homologous to that of the W group, as found using SourceTracker analysis. For the C–F and C–M groups, the values of shared flora were as high as 96% (Figures 2E, F).

### 3.3. Indicators and species (OTU) interactions

**Figure 4:**
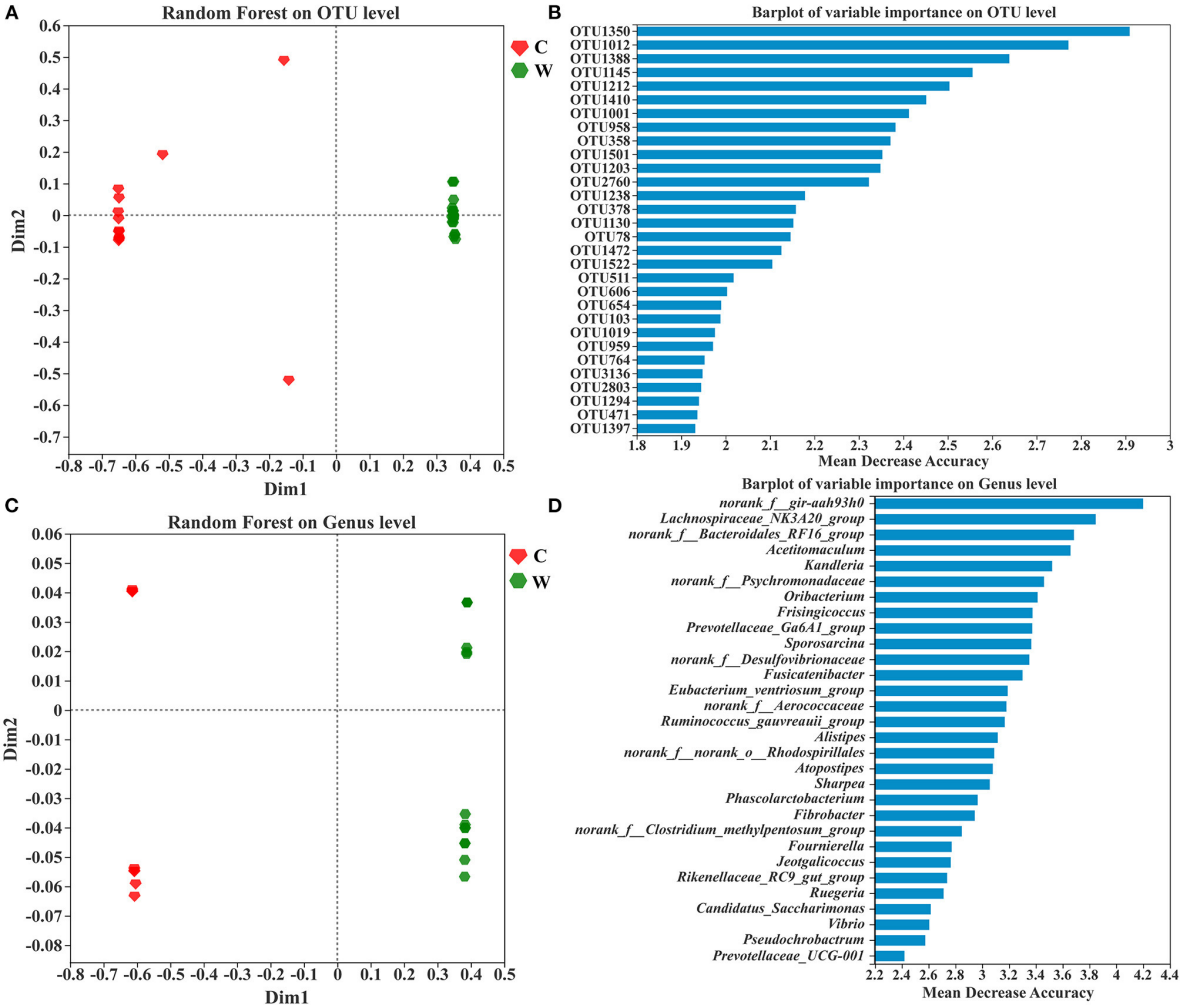
Random forest analysis of sample clustering at OTU **(A)** and genus levels **(C)**, and the top 30 OTUs **(B)** and genus **(D)** with variable importance based on their mean decrease in accuracy.

The samples exhibited significant groupings based on the random forest at OTU and genus levels, indicating habitat specificity between both groups (Figures 4A, C). The top 30 taxa of variable importance from random forest-based prediction at OTU and genus levels are shown in Figures 4B, D. Mean decrease accuracy was used as a measure of variable importance. OTU1350, OTU1012, and OTU1388 exhibited the top three taxa of variable importance in the random forest model, and these belonged to the phyla Firmicutes and Proteobacteria, to which the top three families also belong (Supplementary Figure 3). However, the top three genera in the ranking are from the phyla Firmicutes and Bacteroidetes.

To identify the characteristics and categories of the gut microbiota between all groups, the R package function Indval was used to seek indicators showing significant differences (*p* < 0.05). Here, indicator species (OTUs) can signal changes in the gut microbiota of captive and wild Alpine musk deer and, therefore, can serve as a proxy to characterize these changes. There were 166 indicators with higher richness (threshold value: 0.75–1.0) in the C group, mainly belonging to Firmicutes (31.93%), Bacteroidetes (18.67%), Proteobacteria (27.11%), Actinobacteriota (7.23%), and Verrucomicrobiota (4.22%). However, there were only 53 indicators in the W group, which were classified as Firmicutes (60.38%), Actinobacteriota (18.87%), Proteobacteria (13.21%), Desulfobacterota (3.77%), and Bacteroidetes (3.77%) (Supplementary Table 1). The C and W groups shared the same category and number (33) of indicators with a medium abundance (threshold value: 0.25–0.75) in significant differences (Supplementary Table 2). By contrast, the rare species indicators with a low relative abundance (threshold value: 0–0.25) exhibited extreme differences between the W and C groups, and no indicators were shared by the two groups. The number of rare species indicators (indicators with a significant difference and low relative abundance) in the W group (69) was much higher than that in the C group (43) and mostly belonged to the phyla Firmicutes and Bacteroidetes (Supplementary Table 3). For the captive sex group, the unique indicators in the C–F group contained *Gordonibacter*, norank_f_norank_o_Microtrichales, ynechococcus_CC9902, Lachnospiraceae_UCG-008, and *Acidibacter*, which belong to the phyla Actinobacteriota, Firmicutes, and Proteobacteria (Supplementary Figure 4).

**Figure 5:**
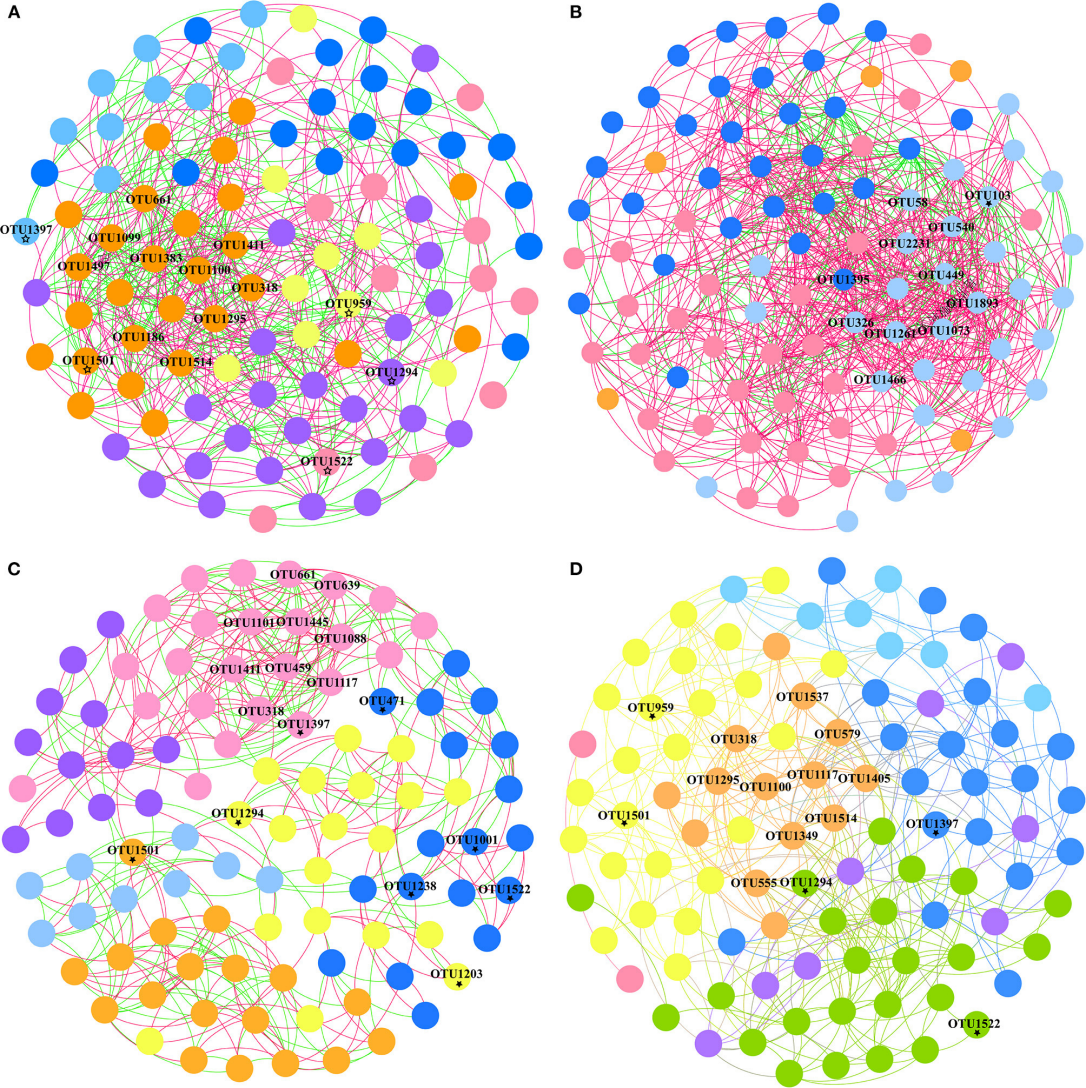
Network modules and connectivity of OTUs in different groups. Co-occurrence networks of OTUs present different modules in captive **(A)**, wild **(B)**, captive female **(C)**, and captive male groups **(D)** with positive and negative correlations (top 30 OTUs derived from random forest results indicated by ⋆).

OTU-level co-occurrences were also represented by Spearman's correlations (|*r*| > 0.5, *p* < 0.05), and the networks among species (OTUs) only incorporated taxa under different environments and sexes. The networks in the W group contained 858 edges with positive and negative correlation proportions of 75.29 and 24.71%, respectively, which was considerably more complex than the 624 edges in the C group with positive and negative correlation proportions of 53.85 and 46.15%, respectively (Figures 5A, B, Supplementary Table 4). Modules V and VI were lacking in the W group (modularity = 0.326), and hub OTUs (top 10) with higher connectivity were predominantly scattered in module I, while it is module IV in group C (modularity = 0.327). All hub OTUs (top 10) between C and W groups are in the phylum Firmicutes. The layout of the functional modules varies between sexes in the captive group. Hub OTUs (top 10) are reflected in modules III and IV, respectively (Figures 5C, D). Most hub OTUs in both groups (C–F and C–M) belonged to Firmicutes, but OTU445 (in the C–F group), OTU1537, and OTU579 (in the C–M group) were derived from the phylum Bacteroidetes. Meanwhile, hub OTUs 1445 (in the C–F group) and 579 and 555 (in the C–M group) belong to Alistipes, Rikenellaceae_RC9_gut_group, and Atopostipes, respectively, which is consistent with the results obtained using random forest. Some of the top 30 OTUs were derived from random forest results, which were distributed in different modules, and exhibited a certain network relationship (Figure 5).

### 3.4. Functional prediction of gut microbiota

**Figure 6:**
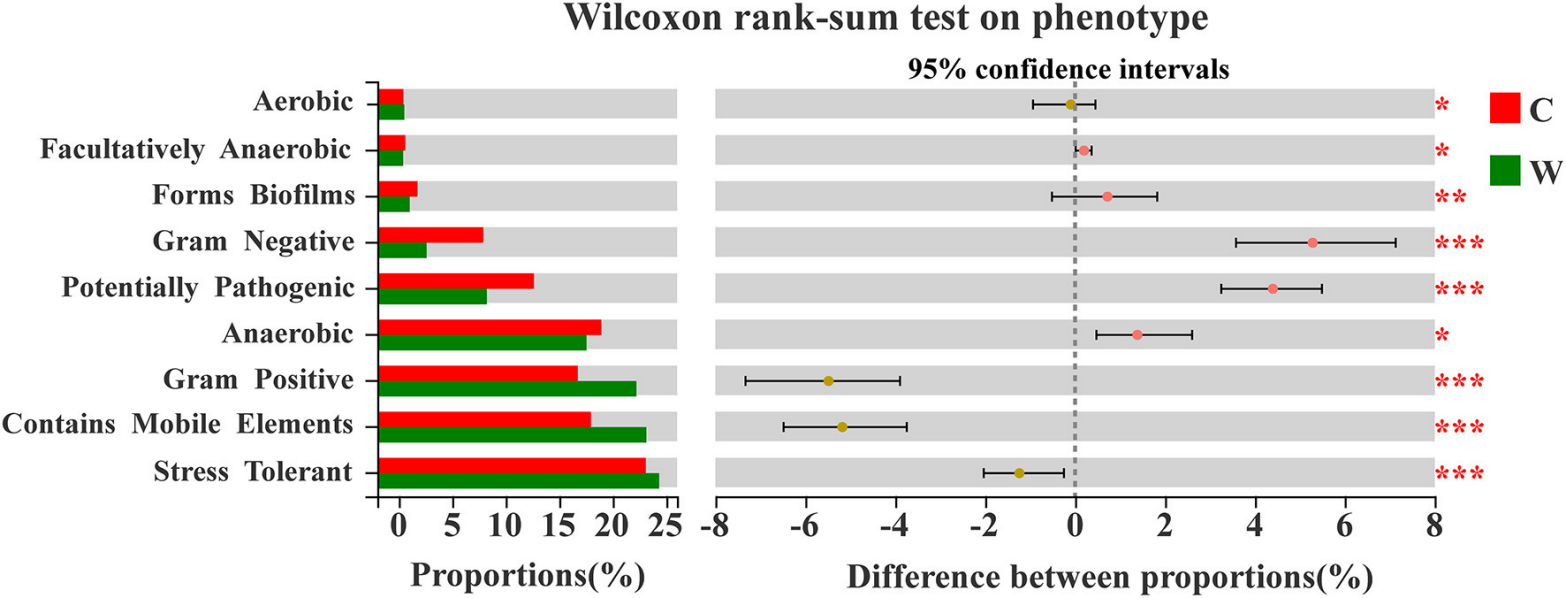
Enterotype by Wilcoxon rank-sum test based on Bug Base in both groups (W: wild, C: captive; significance *p < 0.05, **p < 0.01, and ***p < 0.001).

A Wilcoxon rank-sum test on the phenotype in both groups revealed a significant difference between proportions based on BugBase (Figure 6). The phenotypes with a relative contribution of more than 10% were stress-tolerant, Gram-positive, anaerobic, potentially pathogenic, and contain mobile elements, thus playing an important role in the adaptation of the microbial community to their respective environmental state. Stress tolerance was dominant in both groups, and the W group had a higher relative contribution from the families Lachnospiraceae, Oscillospiraceae, and UCG-010 belonging to the phylum Firmicutes, whereas the family Rikenellaceae in the phylum Bacteroidetes had a higher relative contribution of this phenotype in the C group (Supplementary Figure 5).

**Figure 7:**
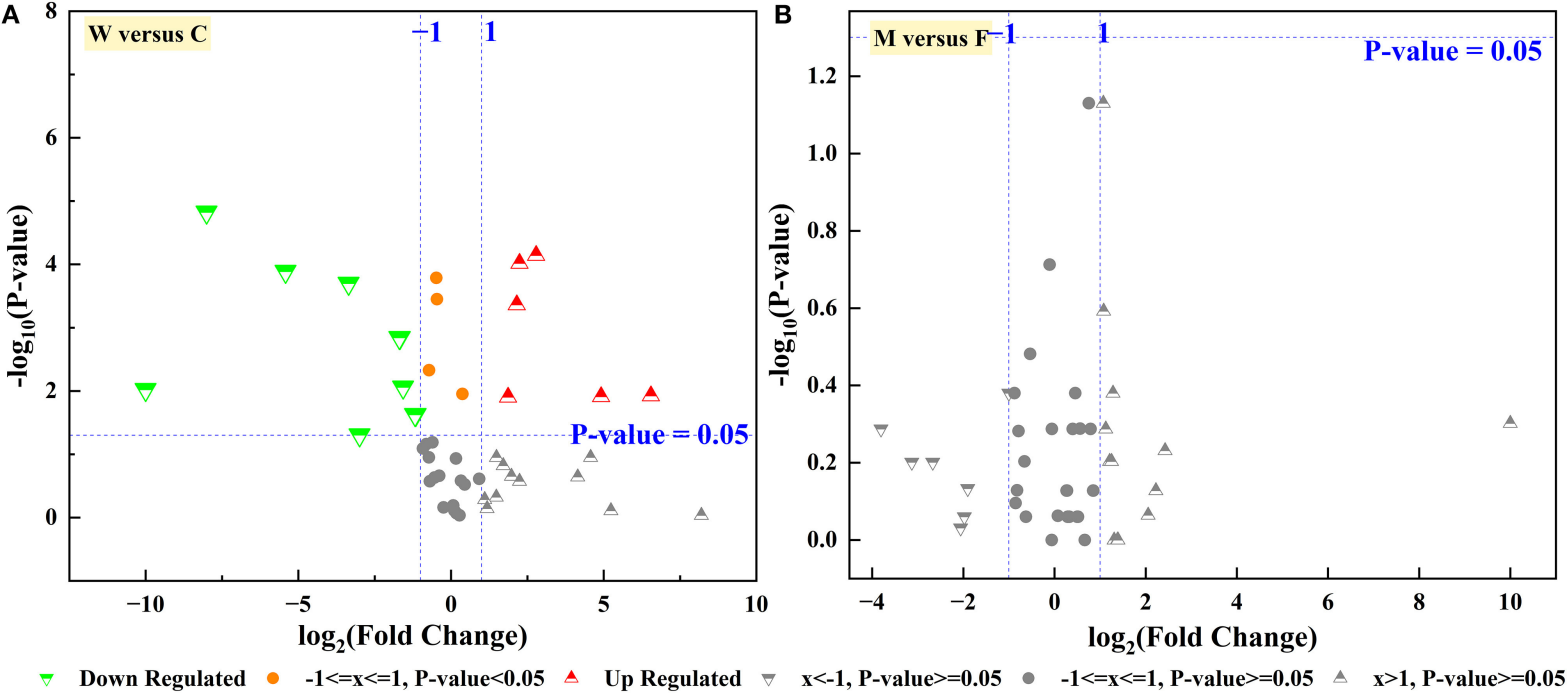
Volcano plot gut microbiota function of Alpine mush deer. **(A)** Captive group (C) vs. wild group (W), **(B)** female group (F) vs. male group (M) in captivity.

FAPROTAX and the Wilcoxon rank-sum test were employed to predict the functions of the identified microbial community. As shown in Figure 7A, a relatively higher proportion of chemoheterotrophy and fermentation was observed in the C group than in the W group, which might relate to the diet and energy supply of the deer. In addition, the functions with a relatively higher proportion and significant difference in the W group compared with the C group were animal parasites or symbionts, mammal gut, and human gut (Table 1). This indirectly reflects the fact that captive animals experience less stress and disease encounters owing to proactive health testing by managers, while wild animals are more resistant to disease. Moreover, in this study, no significant differences in enterotype and ascending functions were observed between captive male and female Alpine musk deer (Figure 7B).

**Table 1:** Predicted composition of functional groups by FAPROTAX and Wilcoxon rank-sum test in captive (C) and wild (W) groups.

**Functional groups**	**Log_2_ (fold change)**	***p*-value**	**Significance**
Reductive acetogenesis	−8.0046	1.500 × 10^−5^	Down
Mammal gut	2.7863	7.220 × 10^−5^	Up
Human gut	2.7863	7.220 × 10^−5^	Up
Animal parasites or symbionts	2.2436	9.640 × 10^−5^	Up
Manganese oxidation	−5.4184	1.289 × 10^−4^	Down
Chemoheterotrophy	−0.4792	1.630 × 10^−4^	Down
Cellulolysis	−3.3564	1.998 × 10^−4^	Down
Fermentation	−0.4614	3.545 × 10^−4^	Down
Dark thiosulfate oxidation	2.1542	4.359 × 10^−4^	Up
Nitrite denitrification	−1.6761	1.434 × 10^−3^	Down
Nitrous oxide denitrification	−1.6761	1.434 × 10^−3^	Down
Nitrate denitrification	−1.6761	1.434 × 10^−3^	Down
Denitrification	−1.6929	1.434 × 10^−3^	Down
Aerobic chemoheterotrophy	−0.7193	4.693 × 10^−3^	Down
Iron respiration	−1.5674	8.723 × 10^−3^	Down
Manganese respiration	−10	9.497 × 10^−3^	Down
Intracellular parasites	0.3716	1.113 × 10^−2^	Up
Human pathogens meningitis	6.5452	1.198 × 10^−2^	Up
Ligninolysis	4.9141	1.227 × 10^−2^	Up
Human pathogens all	1.8693	1.254 × 10^−2^	Up
Nitrate respiration	−1.1565	2.367 × 10^−2^	Down
Hydrocarbon degradation	−1.1811	2.367 × 10^−2^	Down
Thiosulfate respiration	−2.9937	4.940 × 10^−2^	Down

## 4. Discussion

Gut microbiota, an important “acquired organism,” have a marked impact on the survival and fitness of the host and are involved in many important physiological processes. The survival status of animals under various conditions is easily obtained without harm to the animals through feces sampling and 16S rRNA high-throughput sequencing (Aguirre and Venema, 2015; Rounge et al., 2018; Antwis et al., 2019). Therefore, this approach is widely applied to maintain and restore rare and endangered wildlife populations (Wang et al., 2016; Thitaram and Brown, 2018; Ning et al., 2020). In this study, a series of statistical tests and machine learning approaches were employed to obtain new data on the gut microbiota of captive and wild Alpine musk deer based on fecal samples. Alpine musk deer of captive populations were found to have higher gut microbial diversity (alpha and beta diversities) than wild populations, suggesting that captive populations have complex and stable gut microbiota and have adapted well to local habitats. These results are different from those of a previous study on gut microbiota between captive and wild forest musk deer, where there was only a significant difference in beta diversity and no significant difference in alpha diversity (Li et al., 2017). Various factors affect the gut microbiota in mammals, including diet, which is the main and most important source of energy and nutrition for both the host and the gut microbiota (Ley et al., 2008; Wu et al., 2011; Navarrete et al., 2012; Hasebe et al., 2022). Numerous studies have shown decreasing community similarity with increasing differences between habitats and species (Liu et al., 2021; Barrionuevo et al., 2022; Yang et al., 2022), and that animal sex is also a predominant factor affecting the gut microbiota (Zhang et al., 2022). Although the direct analysis method did not reflect the differences in gut microbiota by sex (Supplementary Figure 2B), significant differences were revealed by using a supervised analysis method (PLS-DA) (Figure 2). In general, captive animals are limited by the freedom of living space and food, but their food sources are adequate and balanced. In this study, both local isolated populations (captive and wild populations) survive in the same latitudinal zone and have similar climatic conditions. The diet of the captive Alpine musk deer mainly consisted of fresh leaves combined with foods having high protein and carbohydrate contents, while the wild Alpine musk deer predominantly fed on wild plant leaves and fungi with high crude fiber and ether extract. Thus, differences in gut microbiota between captive and wild Alpine musk deer might be related to the difference in the environment and access to food.

Community assembly mechanisms—an important ecological aspect driving forces that shape the community—are determined by a combination of deterministic and stochastic ecological processes (Stegen et al., 2012; Dini-Andreote et al., 2015). Our results showed that the assembly processes of gut microbial communities in captive and wild Alpine musk deer were mostly deterministic. Heterogeneous selection (βNTI ≥ +2) was the predominant deterministic process, which contains ecological selection by abiotic environmental factors (environmental filtering) and mutual antagonism and synergism between species (Vellend, 2010). For captive and wild Alpine musk deer, significant differences in foods result in different habitat preferences and adaptations of gut microbiota under similar climatic conditions, contributing to this assembly process, which can be explained through a weak influent immigration influence and strong environmental filtering. Sex also influences the community assembly process, which is consistent with the effect of sex on gut microbial diversity. Network analysis and co-occurrence patterns revealed strong and complex species (OTUs) interactions and indicators (Toju et al., 2016), highlighting the importance of interspecific interactions in the community assembly process. The indicators with higher richness in both groups predominantly belong to the phylum Firmicutes, which is commonly found in the gut microbiota in ruminants (Gruninger et al., 2014; Ishaq and Wright, 2014; Li et al., 2017). Indicators with low richness in both groups also demonstrated significant differences, and their low abundance but large numbers, which were mostly derived from the phyla Firmicutes and Bacteroidetes, illustrates their subtle role in the host gut. Bacteroidetes are another component of the gut microbiota present in ruminants (Sundset et al., 2007; Gruninger et al., 2014; Li et al., 2017).

Although the correlation network of the wild Alpine musk deer had two fewer functional modules than the captive group at the OTU level, it showed a stronger positive and negative correlation to evaluate intestinal health. The hub OTUs (top 10) with higher connectivity are presented inside each group. For instance, in the captive group, OTU661 and OTU1295 belonged to the family Lachnospiraceae, which participates in fermentation to produce acetic acid and butyric acid, the main source of energy for the host, and aids in the prevention of colon cancer (Dahiya et al., 2019). OTUs 1411, 1100, 1186, and 1514 were from the family Eubacterium_coprostanoligenes_group, which has beneficial effects on dyslipidemia (Wei et al., 2021). OTU1497 belonged to the family Christensenellaceae, the members of which are associated with immune regulation and healthy homeostasis and are widely found in human and animal intestines and mucous membranes (Kong et al., 2016). For the wild group, OTUs 2231, 1073, and 1466 were from the family Lachnospiraceae, while OTU540 belonged to the family Eubacterium_coprostanoligenes_group. UCG-010 is a family shared between the captive (OTU1383) and wild (OTUs 58, 1893, 1262, and 326) groups. However, all hub OTUs in both groups belonged to the phylum Firmicutes, which can degrade cellulose into volatile fatty acids for the host to use. The network analysis showed that both populations of Alpine musk deer had relatively good health, and although the intestinal microbes of the deer differed in different living environments, their complex relationships indicated participation in the energy supply process of the host. The correlation network of the captive male Alpine musk deer had one more functional module than that of the female group, but the correlations within the groups were essentially similar, and all hub OTUs belonged to the phyla Firmicutes and Bacteroidetes. Bacteroidetes also degrade carbohydrates and proteins and are vital for improving host immunity and maintaining the balance of gut microbiota (Hooper et al., 2001; Bäckhed et al., 2004; Hooper, 2004; Sears, 2005).

Functional predictions in our study were performed in parallel using BugBase and FAPROTAX, which have powerful algorithms and novel databases (Louca et al., 2016; Zhang et al., 2019). The phenotypes of stress tolerance and contains mobile elements were two relatively important players, and the wild group was significantly higher than the captive group. Meanwhile, the microbial families between these two phenotypes and network interactives described earlier were consistent. We speculate that Alpine musk deer require more diverse intestinal phenotypes to aid or supplement metabolic needs in the extreme survival environment in the wild. Owing to concentrated captive breeding, captive populations also exhibit a significantly higher proportion of phenotypes of potentially pathogenic bacteria, despite health management. The relatively higher abundance of the function of animal parasites or symbionts in the wild population may favor host gut homeostasis and response to environmental changes. It was previously reported that the energy intake capacity of a wild group was better than that of a captive group, referring to a higher Firmicutes/Bacteroidetes (F/B) ratio that may be associated with the increased energy harvest from colonic fermentation (Sun et al., 2020). However, function prediction of the gut microbiota showed that fermentation of the captive group was relatively higher than that of the wild group in our study, which does not indicate a contradiction with our previous findings. The function of gut microbiota in the wild group may be more tilted or involved in other aspects such as the human gut and mammal gut.

## 5. Conclusion

The results obtained in this study enhance the understanding of microbial ecology in ruminants derived from captive and wild environments. This study was focused on the gut microbial community assembly process of Alpine musk deer in different environments, with the aim of revealing the state of adaptation under the presence of direct environmental changes. There were significant differences in gut microbial diversity and structure among both groups, and the microbial community assembly was largely driven by deterministic mechanisms. The heterogeneous selection was the main deterministic process, which might be closely related to food obtained under similar climatic conditions. Although the relative abundance of indicators showed significant differences at each level, their origin was ultimately attributed to the phyla Firmicutes, Bacteroidetes, and Proteobacteria. There was a higher positive correlation in the wild group, and the captive group showed significant differences in the function of chemoheterotrophy and fermentation compared with the wild group, but the opposite was observed for the functions of animal parasites or symbionts, which might be linked to the diet, energy supply, and healthcare of the animals. Our study also suggested sex was an important factor in the deterministic process of microbial community assembly, but the quantitative and correlated relationships between influential factors (e.g., environment, food, and sex) and gut microbiota warrant further investigation in future.

## Data availability statement

Publicly available datasets were analyzed in this study. This data can be found at: NCBI/BioProject/PRJNA380002 (https://www.ncbi.nlm.nih.gov/bioproject/PRJNA380002).

## Ethics statement

The animal study was reviewed and approved by the Board of Ethical Committee for Experimental Animals, Northeast Forestry University.

## Author contributions

ZZ: conceptualization, methodology, visualization, sample collection, supervision, and writing the original draft. MD: conceptualization, methodology, visualization, and data curation. YS: investigation and sample collection. RHK: investigation and writing, reviewing, and editing. JC: visualization. LT: supervision, investigation, funding acquisition, writing, reviewing, and editing. ZL: conceptualization, supervision, investigation, and funding acquisition. All authors contributed to the manuscript and approved the submission.

## Footnotes

^1^ https://cloud.majorbio.com/page/tools/

^2^ http://bugbase.cs.umn.edu
