# Supplementary material for: Different living environments drive deterministic microbial community assemblages in the gut of Alpine musk deer (Moschus chrysogaster)
Source: Front Microbiol. 2023 Jan 13;13:1108405. doi: 10.3389/fmicb.2022.1108405 (PMC9880224; doi:10.3389/fmicb.2022.1108405)
Supplement: Supplementary file 4 [file Data_Sheet_1.docx]

Supplementary Material

Different Living Environments Drive Deterministic Microbial Community Assemblages in the Gut of Alpine Musk Deer (*Moschus chrysogaster*)

Zhirong Zhang^1^, Mengqi Ding^2^, Yujiao Sun^1,3^, Romaan Hayat Khattak^1^, Junda Chen^1^, Liwei Teng^1,4*^ and Zhensheng Liu^1,4*^

^1^ College of Wildlife and Protected Areas, Northeast Forestry University, Harbin, China

^2^ State Key Laboratory of Urban Water Resource and Environment, School of Environment, Harbin Institute of Technology, Harbin, China

^3^ College of Food and Biological Engineering, Henan University of Animal Husbandry and Economy, Zhengzhou, China

^4^ Key Laboratory of Conservation Biology, National Forestry and Grassland Administration, Harbin, China

*** Correspondence:** Liwei Teng: tenglw1975@163.com; Zhensheng Liu: zhenshengliu@163.com

# Supplementary Figures and Tables

## Supplementary Figures


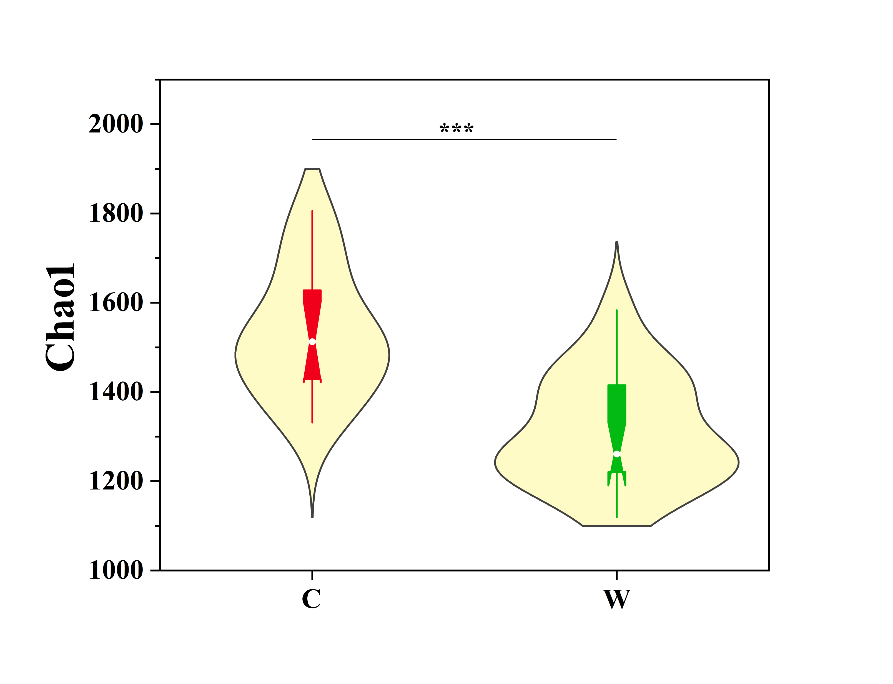


**Supplementary Figure 1.** Comparison of Chao indices of gut microbiota of the captive (C) and wild (W) alpine musk deer.


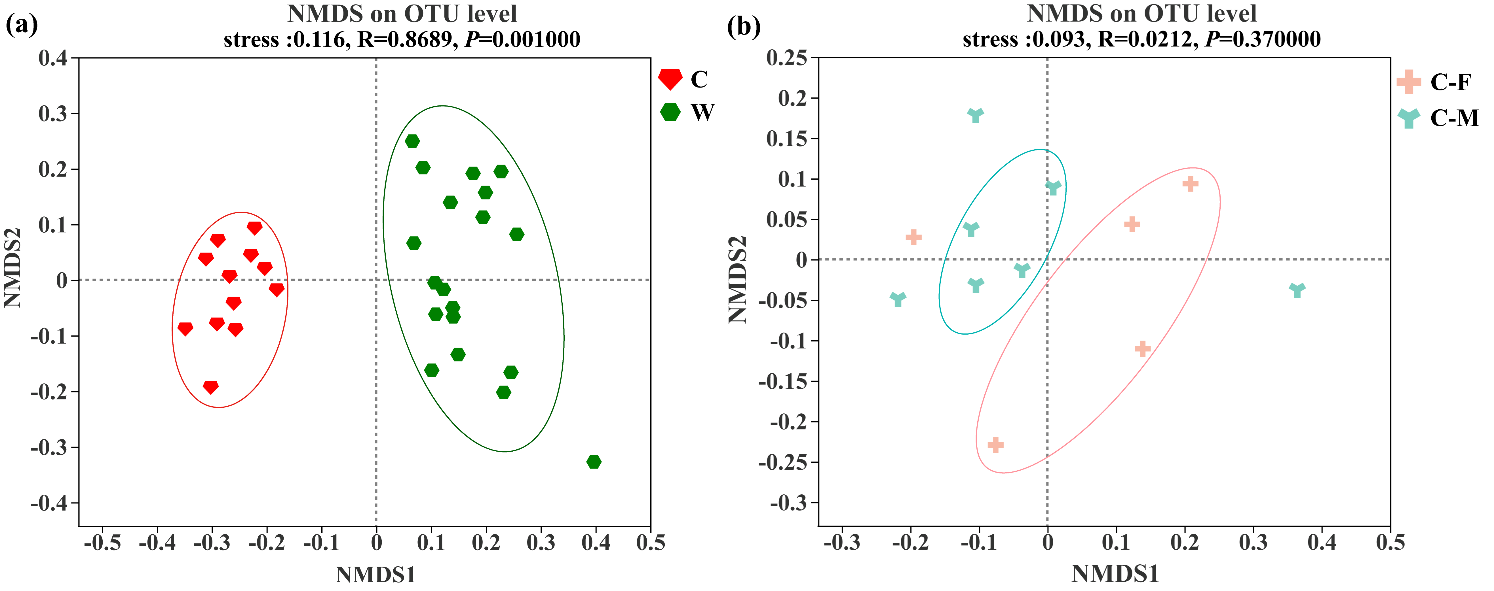


**Supplementary Figure 2.** Nonmetric multidimensional scaling (NMDS) on OTU level based on Bray-Curtis dissimilarities revealed separation of gut microbiota of alpine musk deer in different habitat. (a) Gut microbiota of alpine musk deer in captivity (C) and wild (W), (b) gut microbiota of captive alpine musk deer between female (C-F) and male (C-M).

**
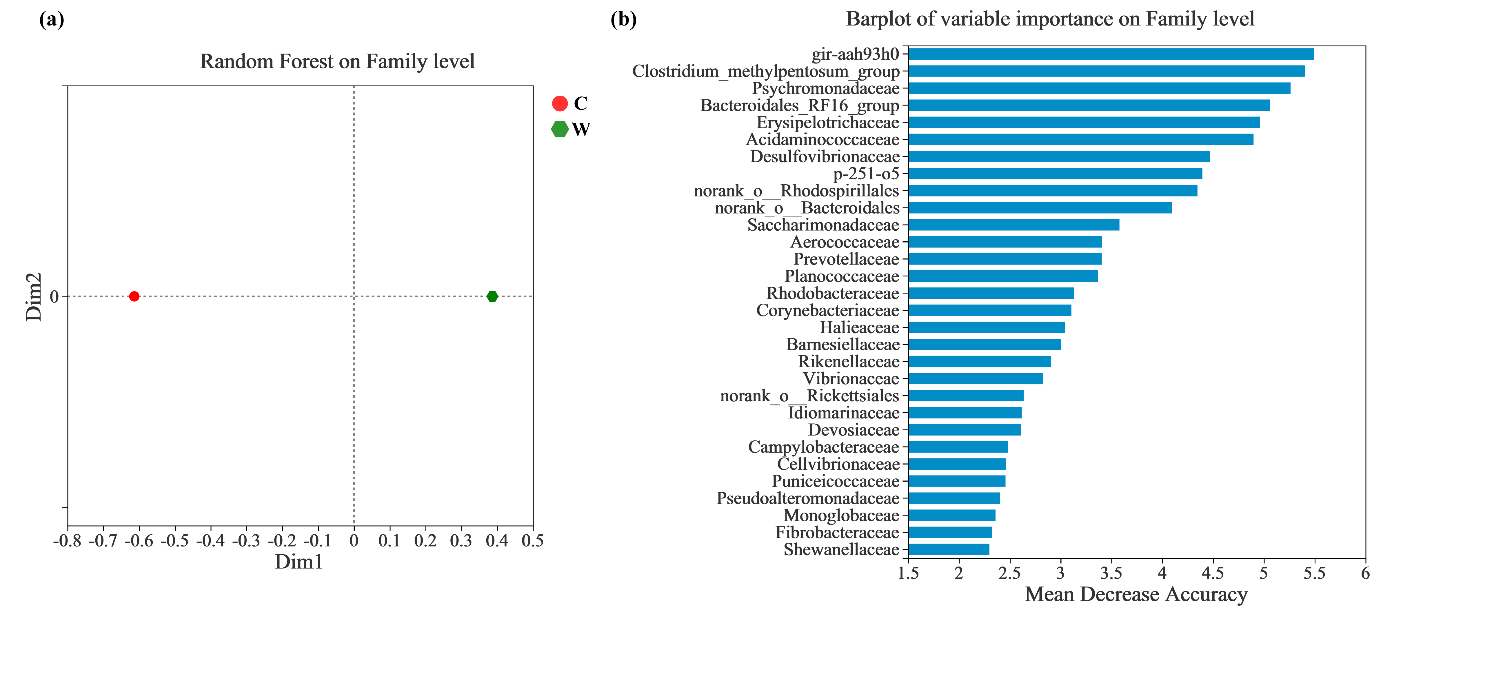
**

**Supplementary Figure 3.** Random forest analysis of sample clustering at family (a) and the top 30 family (b) with variable importance based on their mean decrease in accuracy.


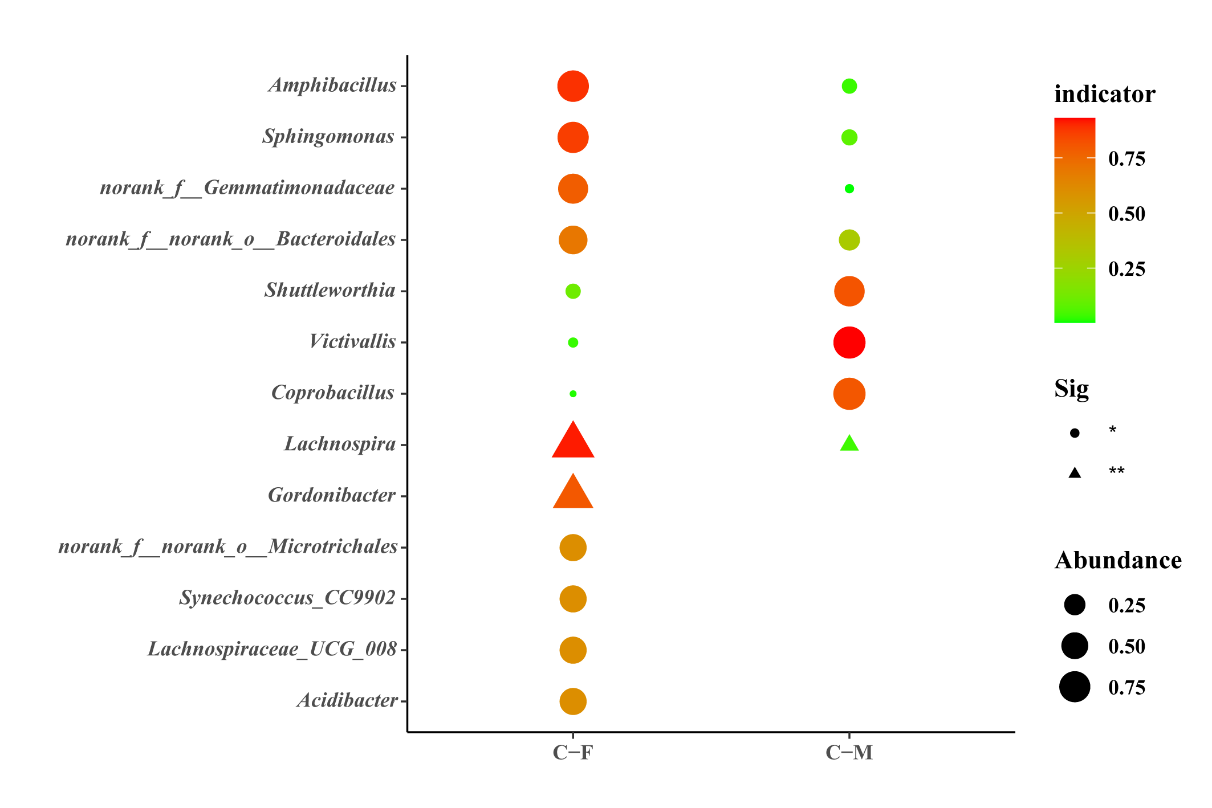


**Supplementary Figure 4.** The indicators used Indval Function analysis showed significant difference of gut microbiota in captive alpine musk deer between female (C-F) and male (C-M) groups.


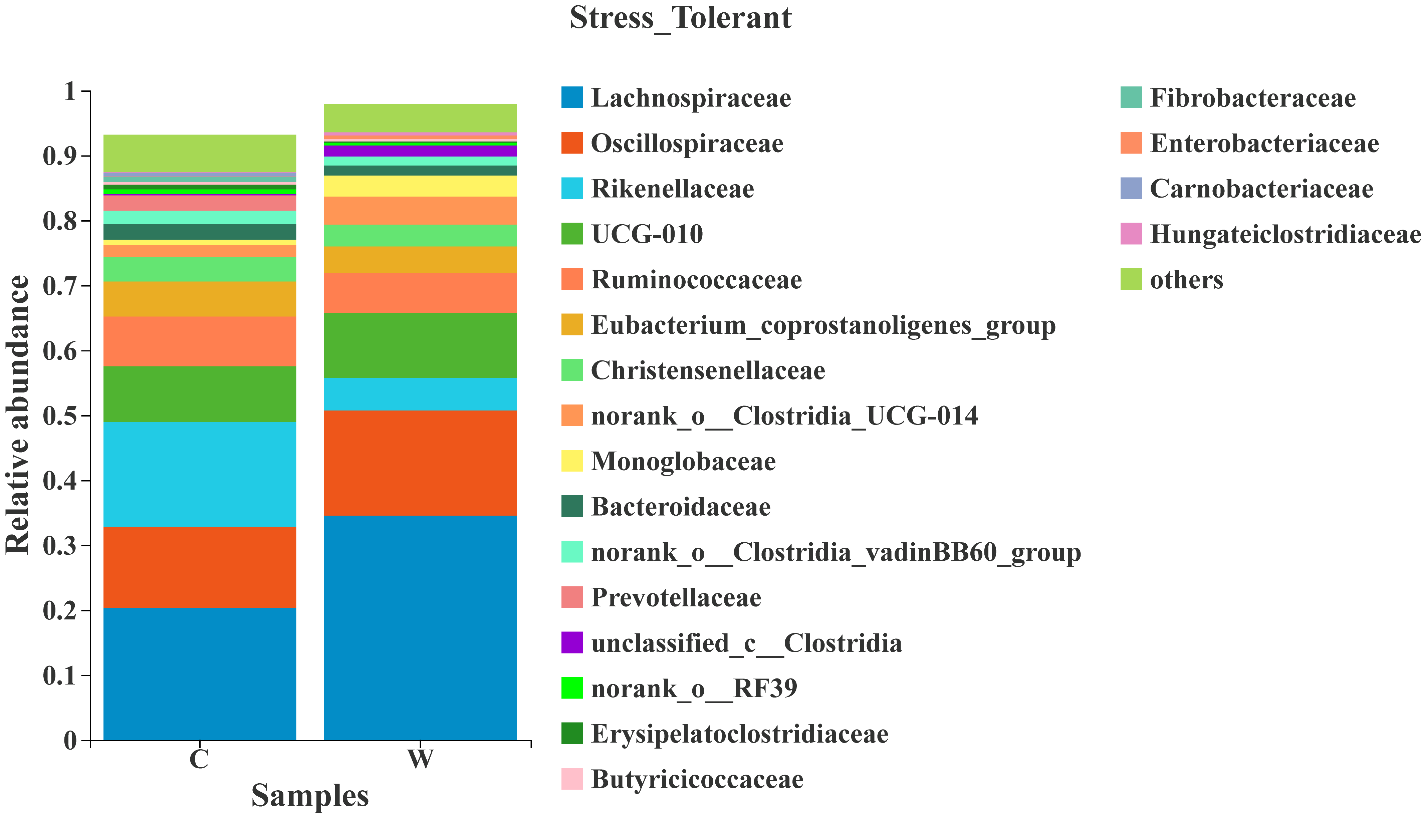


**Supplementary Figure 5.** The relative abundance of gut microbiota based on enterotype of Stress tolerant in both captive (C) and wild (W) groups.

## Supplementary Tables

**Supplementary Table 4.** Co-occurrence networks results for the gut microbial community of alpine musk deer derived from wild (W) and captive (C) groups both female (C-F) and male (C-M).

| **Group** | **Co-occurrence networks parameters** | | | | | | |
| --- | --- | --- | --- | --- | --- | --- | --- |
|  | **Nodes** | **Edges** | **Positive** | **Negative** | **Diameter** | **Modularity** | **Clustering coefficient** |
| W | 100 | 858 | 75.29 | 24.71 | 5 | 0.326 | 0.509 |
| C | 100 | 624 | 53.85 | 46.15 | 7 | 0.327 | 0.441 |
| C-F | 100 | 436 | 54.13 | 45.87 | 8 | 0.644 | 0.590 |
| C-M | 100 | 412 | 51.7 | 48.3 | 7 | 0.522 | 0.511 |
